# The malaria testing and treatment landscape in Benin

**DOI:** 10.1186/s12936-017-1808-x

**Published:** 2017-04-26

**Authors:** ACTwatch Group, Louis Akulayi, Angela Alum, Andrew Andrada, Julie Archer, Ekundayo D. Arogundade, Erick Auko, Abdul R. Badru, Katie Bates, Paul Bouanchaud, Meghan Bruce, Katia Bruxvoort, Peter Buyungo, Angela Camilleri, Emily D. Carter, Steven Chapman, Nikki Charman, Desmond Chavasse, Robyn Cyr, Kevin Duff, Gylsain Guedegbe, Keith Esch, Illah Evance, Anna Fulton, Hellen Gataaka, Tarryn Haslam, Emily Harris, Christine Hong, Catharine Hurley, Whitney Isenhower, Enid Kaabunga, Baraka D. Kaaya, Esther Kabui, Beth Kangwana, Lason Kapata, Henry Kaula, Gloria Kigo, Irene Kyomuhangi, Aliza Lailari, Sandra LeFevre, Megan Littrell, Greta Martin, Daniel Michael, Erik Monroe, Godefroid Mpanya, Felton Mpasela, Felix Mulama, Anne Musuva, Julius Ngigi, Edward Ngoma, Marjorie Norman, Bernard Nyauchi, Kathryn A. O’Connell, Carolyne Ochieng, Carolyne Ogada, Linda Ongwenyi, Ricki Orford, Saysana Phanalasy, Stephen Poyer, Justin Rahariniaina, Jacky Raharinjatovo, Lanto Razafindralambo, Solofo Razakamiadana, Christina Riley, John Rodgers, Andria Rusk, Tanya Shewchuk, Simon Sensalire, Julianna Smith, Phok Sochea, Tsione Solomon, Raymond Sudoi, Martine Esther Tassiba, Katherine Thanel, Rachel Thompson, Mitsuru Toda, Chinazo Ujuju, Marie-Alix Valensi, Vamsi Vasireddy, Cynthia B. Whitman, Cyprien Zinsou, Cyprien Zinsou, Adjibabi Bello Cherifath

**Affiliations:** 1 Population Services International, 1120 19th St NW Suite 600, Washington, DC 20036 USA; 2 Association Beninoise pour le Marketing Social, Lot 919 Immeuble Montcho, Sikecodji, Cotonou, Republic of Benin; 3 Programme National de Lutte contre le Paludisme, Ministère de la Santé, Cotonou, Benin

**Keywords:** Benin, Malaria case management, Private sector, Public sector, Artemisinin-based combination therapy, Diagnostic test, ACT subsidy

## Abstract

**Background:**

Since 2004, artemisinin-based combination therapy (ACT) has been the first-line treatment for uncomplicated malaria in Benin. In 2016, a medicine outlet survey was implemented to investigate the availability, price, and market share of anti-malarial treatment and malaria diagnostics. Results provide a timely and important benchmark to measure future interventions aimed at increasing access to quality malaria case management services.

**Methods:**

Between July 5th to August 6th 2016, a cross sectional, nationally-representative malaria outlet survey was conducted in Benin. A census of all public and private outlets with potential to distribute malaria testing and/or treatment was implemented among 30 clusters (arrondissements). Outlets were eligible for inclusion in the study if they met at least one of three study criteria: (1) one or more anti-malarials reportedly in stock on the day of the survey; (2) one or more anti-malarials reportedly in stock within the 3 months preceding the survey; and/or (3) provided malaria blood testing. An audit was completed for all anti-malarials, malaria rapid diagnostic tests (RDT) and microscopy.

**Results:**

7260 outlets with the potential to sell or distribute anti-malarials were included in the census and 2966 were eligible and interviewed. A total of 17,669 anti-malarial and 494 RDT products were audited. Quality-assured ACT was available in 95.0% of all screened public health facilities and 59.4% of community health workers (CHW), and availability of malaria blood testing was 94.7 and 68.4% respectively. Sulfadoxine–pyrimethamine (SP) was available in 73.9% of public health facilities and not found among CHWs. Among private-sector outlets stocking at least one anti-malarial, non-artemisinin therapies were most commonly available (94.0% of outlets) as compared to quality-assured ACT (36.1%). 31.3% of the ACTs were marked with a “green leaf” logo, suggesting leakage of a co-paid ACT into Benin’s unsubsidized ACT market from another country. 78.5% of the anti-malarials distributed were through the private sector, typically through general retailers (47.6% of all anti-malarial distribution). ACT comprised 44% of the private anti-malarial market share. Private-sector price of quality-assured ACT ($1.35) was three times more expensive than SP ($0.42) or chloroquine ($0.41). Non-artemisinin therapies were cited as the most effective treatment for uncomplicated malaria among general retailers and itinerant drug vendors.

**Conclusions:**

The ACTwatch data has shown the importance of the private sector in terms of access to malaria treatment for the majority of the population in Benin. These findings highlight the need for increased engagement with the private sector to improve malaria case management and an immediate need for a national ACT subsidy.

**Electronic supplementary material:**

The online version of this article (doi:10.1186/s12936-017-1808-x) contains supplementary material, which is available to authorized users.

## Background

In Benin, important gains in malaria control have been achieved in recent years, however, malaria remains a leading cause of morbidity and mortality. In 2015, the World Health Organization (WHO) reported over two million confirmed malaria cases and 1416 deaths in the country [1]. Malaria is cited as the leading reason for medical consultations and hospitalization in Benin [2]. According to population based surveys, only 28% of children under 5 received the first-line treatment for uncomplicated malaria [3] and among pregnant women, only one in four were found to use intermittent treatment as prevention during pregnancy (IPTp) [4]. The financial impact of malaria is also of concern in Benin. It is estimated that households spend approximately one-quarter of their annual income on the prevention and treatment of malaria, meanwhile, 37% of the Benin population live below the poverty line, with a per capita annual income of only $750 [5].

In 2004, the policy for malaria management in Benin changed when the National Malaria Control Programme (NMCP) introduced artemisinin-based combination therapy (ACT), artemether–lumefantrine (AL), for treatment of uncomplicated malaria [1]. Up to that time, chloroquine had been used for first-line therapy against uncomplicated malaria. In 2011, the guidelines changed and stipulated that patients of all ages should receive a confirmatory malaria test prior to treatment. In 2014, updates to national policy brought malarial to case management guidelines further in-line with WHO recommendations and stipulated three doses of SP for IPTp. The NMCP also updated the malarial national case management guidelines to align with the WHO recommendation for treatment of severe malaria with injectable artesunate and injectable artemether [6], though injectable quinine is also still recommended followed by a seven day treatment with oral quinine. Treatment for severe malaria should only be administered at a public or private hospital. Oral artemisinin monotherapies have been banned in Benin since 2008 [1].

As a means to promote universal coverage of first-line treatment and increase rates of confirmatory testing, the NMCP took significant steps to improve malaria case management services across the country. In 2011, public-sector initiatives included free malaria case management to children under 5 years of age and pregnant women. Prior to this, public health facilities had charged fees for consultation, medications, and procedures [7]. The 2014–2018 National Malaria Strategic Plan was also developed and set the goal that by 2030, “…malaria would no longer be a public health problem in Benin” [6]. The strategy aims to decrease the number of annual cases by 75% and reduce the mortality rate to 1 death per 100,000 people.

There has been a substantial increase in the procurement of ACT and malaria rapid diagnostic tests (RDT) as a means to increase universal access to malaria commodities. In 2014, over 1.3 million RDT were procured and in 2015, this increased to almost 1.5 million [1]. A similar pattern followed for the procurement of ACT, which increased from 1.1 million in 2014 to 1.2 million in 2015. Commodities such as ACT and RDT have largely been made available through the public-sector channels.

Other initiatives to improve malaria case management services include expanding access to primary health care services through the training and equipping of community health workers (CHW), including training on the appropriate use of RDT as well as the management of malaria, pneumonia, diarrhoea, and malnutrition [6]. In 2014, it was estimated that over 12,500 CHW were active in the country. Other public-sector initiatives have included funds for the provision of free healthcare to the extremely poor, and the reinforcement of health financing schemes [8].

There have been no major initiatives targeting the private sector in Benin to improve malaria case management services, despite evidence that over 70% of anti-malarials are distributed through this channel [9]. While the national strategy has included the provision of diagnosis, microscopy or RDT, and ACT in selected private health clinics [10], the scale-up is largely in process and has yet to be routinely implemented [6]. Indeed, the private sector in Benin is renowned for being diverse and continuously expanding, with most providers operating informally without a license, mainly because the accreditation process is often perceived as difficult and conveying few benefits [6, 11]. While there is a push to simplify the process by bringing more of the private sector into the formal market, this has yet to be widely implemented.

This lack of private-sector engagement contrasts with several other countries that have benefitted from ACT subsidies aimed to increase access to first-line treatment in the private sector. The most notable of these initiatives was the Affordable Medicines Facility-malaria (AMFm), which continued through 2016 [12, 13] and was implemented in neighbouring Nigeria, as well as seven other countries (Cambodia, Ghana, Kenya, Madagascar, Niger, Uganda, and Tanzania). Through this mechanism, subsidized ACT was available on the market and labelled with a ‘green leaf’ logo to indicate quality-assurance. By increasing quality-assured ACT on the anti-malarial market, the AMFm also aimed to decrease the use of oral artemisinin monotherapies, and non-artemisinin monotherapies, such as chloroquine. Following the AMFm pilot period, the Global Fund continued to support a quality-assured ACT subsidy programme through the Private Sector Co-payment Mechanism (CPM) [14], but Benin was not part of this initiative.

Investigating the anti-malarial and diagnostic market landscape will provide an important benchmark to measure future interventions aimed at increasing access to quality malaria case management services. However, there is limited rigorous evidence on the availability and distribution of anti-malarials and malaria diagnostics in Benin. Since 2008, the multi-country ACTwatch project has been implemented in Benin to fill contemporary evidence gaps by collecting malaria case management commodity market data on anti-malarial medicines, malaria diagnostics, market share, and price in both the private and public sectors [15]. The objective of this paper is to provide practical evidence to inform strategies and policies in Benin towards achieving national malaria control goals, by describing the total market for malaria medicines and diagnostics at the national level according to the most recent survey round. Evidence will point to recommendations for improving coverage of appropriate malaria case management.

## Methods

This was the fourth outlet survey implemented in Benin, with previous surveys conducted in 2009, 2011, and 2014 [16–18]. This study used a cross-sectional, multi-staged cluster sampling approach and was stratified according to urban/rural areas. The outlet survey followed the design implemented in previous survey rounds and across other ACTwatch countries. The outlet survey was implemented from July 5th to August 6th 2016.

### Sampling approach

According to the ACTwatch methodology, outlets are included in the survey if they have the ‘potential’ to sell or distribute anti-malarials. This includes outlets that may not be expected to stock anti-malarial medicines. For example, while public health facilities would be expected to have anti-malarials in stock, the extent to which general retailers or itinerant drug vendors have anti-malarials available may be more debatable. To assess this, the ACTwatch study approach is to include all outlets that could ‘potentially stock’ anti-malarials.

Outlets sampled in Benin’s public sector included public health facilities (including the national referral hospital, regional hospitals, district hospitals, health centers and dispensaries); CHW and private not-for-profit facilities (including non-governmental organisations, hospitals and clinics, and faith-based hospitals and clinics). The private-sector outlet types sampled were private for-profit health facilities (including private hospitals, clinics and diagnostic laboratories); pharmacies (which are registered and licensed by a national regulatory authority); drug stores (Depôts pharmaceutiques); general retailers (grocery stores, kiosks and market stalls selling fast-moving consumer products); and itinerant drug vendors (mobile, unregistered providers selling medicines).

The primary sampling approach taken for ACTwatch outlet surveys entails sampling a set of administrative units (geographic clusters) with a population of approximately 10,000–15,000 inhabitants. The most appropriate administrative unit in Benin matching the desired population size was an ‘arrondissements’. A representative sample of arrondissements was selected using probability proportional to population size sampling, using data from Benin’s fourth Population and Housing census.

As public health facilities, pharmacies, and drug shops (dépôts pharmaceutiques) are important providers of anti-malarials but are relatively uncommon, over-sampling was conducted for these outlet types in Benin. This ‘booster’ sample was obtained by including all public health facilities, pharmacies, and drug shops (dépôts pharmaceutiques) located in the larger administrative area (called a ‘commune’ in Benin) from which a given arrondissement was selected. In this instance, the booster sample covered all public health facilities, pharmacies, and drug shops in the whole commune within which the arrondissements were located.

The sample was stratified by urban–rural ward designation. In total, 15 arrondissement were selected for the main census sample (15 rural, 15 urban). Within each selected arrondissement a census of all outlet types with the potential to provide anti-malarials or diagnostics to consumers was undertaken.

### Eligibility criteria

Outlets were eligible for a provider interview and malaria product audit if they met at least one of three study criteria: (1) one or more anti-malarials reportedly in stock on the day of the survey; (2) one or more anti-malarials reportedly in stock within the three months preceding the survey; and/or (3) provided malaria blood testing (microscopy or RDT). Among eligible outlets, providers were interviewed and all anti-malarials and RDTs were audited.

### Sample size

A series of calculations was completed to identify minimum sample size requirements to detect an increase or decrease in the availability of quality-assured ACT and of malaria blood testing between 2014 and 2016. Calculations examined the sample size required to detect a 20% point change among all outlets, the public sector, the private sector, public health facilities, pharmacies, and general retail outlets.

The required sample size for each research domain (urban and rural areas) was calculated in three steps: (1) determine the required number of anti-malarial-stocking outlets, (2) determine the number of outlets to be enumerated to arrive at this number of anti-malarial-stocking outlets, and (3) determine the number of arrondissement for the census to arrive at this number of outlets.

#### Required number of anti-malarial stocking outlets

The number of anti-malarial-stocking outlets required to detect a change over time is given by:

$$ n = \frac{{deff \times \left[ {Z_{{1{ - }\alpha }} \sqrt {2P\left( {1{ - }P} \right)} + Z_{{1{ - }\beta }} \sqrt {P_{1} \left( {1{ - }P_{1} } \right) + P_{2} \left( {1{ - }P_{2} } \right)} } \right]^{2} }}{{\left( {P_{2} { - }P_{1} } \right)^{2} }} $$

where *n* = desired sample size, P_1_ = the proportion of anti-malarial-stocking outlets with quality-assured ACT/malaria blood testing available in stock in 2014, P_2_ = the expected proportion of anti-malarial-stocking outlets with quality-assured ACT/malaria blood testing available in stock in 2016 (20% point increase or decrease), P = (P_1_ + P_2_)/2, Z_α_ = the standard normal deviation value for an α type I error (two-sided), Z_1 − β_ = the standard normal deviation value for a βtype II error, Deff = the design effect in case of multi-stage arrondissement sample design. Deff figures from the 2014 dataset were used in sample size calculations.

#### Required number of outlets

The estimated number of outlets enumerated needed for the quality-assured ACT availability indicator was determined by the following formula for outlets within urban and rural domains:

$$ {\text{N}} = {{\text{n}} / {\text{P}}}_{{{\text{am}}}}$$

where P_am_ is the proportion of outlets having anti-malarial stocks at the time of the survey among all outlets enumerated. In this equation, the assumptions are as follows: N = desired sample size of all outlets for monitoring availability indicators, n is the number of outlets with anti-malarial stocks at the time of the survey. P_am_ is the proportion of outlets having anti-malarials in stock at the time of the survey among outlets enumerated in 2014 within urban and rural areas. The P_am_ values documented in the 2014 ACTwatch outlet survey were used for 2016 sample size calculations.

#### Required number of arrondissements

The average numbers of outlets by outlet type in arrondissements within urban and rural areas screened during the 2014 outlet survey were used to estimate the number of arrondissements required in 2016 to achieve the desired sample sizes. Considering sample size requirements to detect change over time and average numbers of outlets across each outlet type, the optimal minimum number of localities required to reach desired numbers of outlets was 30 arrondissements (15 urban, 15 rural) plus a booster sample of public health facilities, pharmacies, and drug shops at the commune level.

### Data collection

The outlet survey census involved systematically looking for outlets in each arrondissement and using screening questions to identify outlets for inclusion in the study. Provider interviews and anti-malarial audits were conducted in all eligible outlets, after informed consent procedures. Up to three call-back visits were made to outlets in instances where outlets were closed or providers were not available.

Data were collected using Android phones, except in pharmacies that had a large number of anti-malarial products. In these pharmacies, paper questionnaires were used so that multiple interviewers could audit anti-malarial products simultaneously to shorten the time required to finish the interview. The electronic data collection program was developed using DroidDB (© SYWARE, Inc., Cambridge, MA, USA).

### Measures

Anti-malarial audit information recorded information on the formulation, package size, brand name, active ingredients and strength(s), manufacturer, country of manufacture, reported sale/distribution in the week preceding the survey, retail price, and wholesale price. The RDT audit information collected similar data. In addition to the product audit, a series of questions were administered to the senior-most provider regarding malaria case management knowledge and practices as well as provider training and qualifications.

### Training

Standard ACTwatch tools and training materials were used. A training of trainers was conducted in June 2016 and was followed by a pilot test to evaluate the electronic data collection program. Interviewers, supervisors, and quality controllers then received a training that included an orientation to the study, questionnaire overview, including a focus on how to complete the anti-malarial and RDT audits and how to use the electronic data collection program.

After the training, a field exercise was conducted outside of the selected arrondissements to provide practical experience for the trainees and to evaluate their performance. Supervisors and quality controllers were then chosen from the highest performers in the group, and these candidates then participated in an additional three-day training before the start of data collection. Eight teams were formed, each composed of one supervisor, one quality controller, and five or six interviewers. Representatives from the research agency, Association Beninoise pour le Marketing Social (ABMS), and the ACTwatch central team provided additional supervision and support to the data collection teams in the field for the entirety of the data collection.

### Data analysis

Data collected with paper questionnaires were double entered and verified using a Microsoft Access database. All data cleaning and analysis was completed using Stata 13.1 (©StataCorp, College Station, TX). Sampling weights were applied to account for variations in the probability of selection and standard error estimation accounted for clustering at the arrondissement and commune levels. The sampling weights use for the Benin survey are described in further detail in Additional file 1.

Standard ACTwatch indicators were calculated in line with previous outlet surveys [9, 15, 19]. Anti-malarials were classified as ACT, non-artemisinin therapy, and oral or non-oral artemisinin monotherapy. ACT were further classified as quality-assured ACT or non-quality assured ACT by matching product information to lists of WHO prequalified anti-malarials and Global Fund anti-malarial procurement lists.

Availability of any anti-malarial was calculated with all screened outlets as the denominator. In the public sector, the availability of specific types of anti-malarials was calculated using the denominator of all screened outlets given that anti-malarials should be available at all public health facilities and among CHWs. Availability of specific anti-malarial categories in the private sector was calculated using the total number of private-sector outlets stocking any anti-malarial as the denominator.

Market share was defined as the relative distribution of anti-malarials to individual consumers in the week preceding the survey. In order to allow for meaningful market share comparisons between products, information about anti-malarial distribution was standardized to the adult equivalent treatment dose (AETD). AETD is the amount of active ingredient necessary to treat a 60 kg adult according to WHO treatment guidelines [20]. Volumes distributed were calculated by converting provider reports on the number of anti-malarials sold in the week prior to the survey into AETDs. Volumes were therefore the number of AETDs sold or distributed by a provider in the seven days prior to the survey. All dosage forms were considered when measuring volumes to provide a complete assessment of anti-malarial market share. Public and private-sector booster sample outlets were excluded from market share calculations to avoid over-estimating the role of the private sector.

Median private sector price per AETD was calculated for quality-assured ACT and other non-artemisinin therapies including chloroquine, SP, and quinine. The interquartile range [IQR] was calculated to demonstrate price dispersion. Anti-malarial price was collected in West African Communauté Financière Africaine (CFA) and converted to United States (US) dollars based on official exchange rates for the six-week data collection period.

Provider perceptions regarding the most effective first-line treatment was assessed by administering questions to the senior most provider at all anti-malarial-stocking outlets. Providers were asked to describe what medicine they believed was the most effective treatment for treating uncomplicated malaria in a child and in an adult.

## Results

A total of 7260 outlets were screened for availability of anti-malarials and/or malaria blood testing services. Of screened outlets 2966 met one of the three screening criteria, including 2959 who were stocking anti-malarials on the day of the survey or within the past three months or provided malaria testing. A total of 17,669 anti-malarial and 494 RDT products were audited (Additional file 2).

### Public sector availability

Table 1 shows the availability among all screened public sector outlets. Availability of any anti-malarial was 95.0% among public health facilities and 59.4% among CHWs. Nine in ten public health facilities stocked quality-assured ACT (89.9%) and 54.8% of CHWs. Among public health facilities, availability of the four different package AL pack sizes (6, 12, 18 and 24 tablets) suitable for management of four different weight categories of patients (5–14; 15–24; 25–34 and ≥35 kg) ranged from 48.8 to 65.9% (Additional file 3). Among CHW, 50.4% had AL for children 5–15 kg in stock (a package of six tablets) and availability of other weight/age formulations was less than 5%. SP was available in 73.9% of public health facilities and was not found among CHWs. Oral quinine was available in 87.7% of public health facilities and among 2.3% of CHWs.

**Table 1:** Availability of anti-malarial and malaria blood testing among all public sector outlets screened

	Public health facility	CHW	Total public sector^a^
	% (95% CI)	% (95% CI)	% (95% CI)
Availability of:	**N** **=** **298**	**N** **=** **145**	**N** **=** **536**
Any anti-malarial	95.0 (90.3, 97.4)	59.4 (40.2, 76.1)	72.6 (58.1, 83.5)
Quality-assured ACT	89.9 (83.9, 93.8)	54.8 (32.2, 75.7)	59.4 (43.6, 73.4)
Quality ACT with the ‘green leaf’ logo	3.0 (0.9, 9.8)	0.6 (0.1, 4.3)	4.7 (2.0, 10.8)
Sulfadoxine–pyrimethamine	73.9 (63.9, 81.9)	0.0 (–)	20.6 (14.9, 27.7)
Oral quinine	87.7 (82.7, 91.3)	2.3 (0.4, 11.7)	33.2 (25.0, 42.6)
Chloroquine	0.4 (0.1, 1.6)	0.0 (–)	1.3 (0.5, 3.5)
Oral artemisinin monotherapy	0.0 (–)	0.0 (–)	0.0 (–)
Artesunate injection	5.3 (1.6, 16.6)	0.0 (–)	1.3 (0.4, 4.1)
Artemether injection	6.2 (2.1, 16.7)	0.0 (–)	5.5 (2.5, 11.8)
Quinine injection	79.3 (69.5, 86.5)	4.5 (0.8, 22.1)	32.2 (23.9, 41.9)
Availability of:	**N** **=** **298**	**N** **=** **145**	**N** **=** **536**
Any diagnostic test	94.7 (90.1, 97.2)	68.4 (47.3, 84.0)	69.4 (56.9, 79.6)
Microscopy	28.7 (21.4, 37.2)	0.9 (0.1, 5.1)	8.9 (6.0, 13.0)
RDT	94.4 (89.8, 96.9)	68.4 (47.3, 84.0)	68.3 55.8, 78.7)
Readiness for malaria case management:	**N** **=** **298**	**N** **=** **145**	**N** **=** **536**
Quality-assured ACT and malaria testing available	89.0 (82.7, 93.2)	49.7 (28.4, 71.2)	53.7 (39.2, 67.6)
Quality-assured ACT no malaria testing available	1.0 (0.2, 3.6)	5.1 (2.3, 11.0)	5.7 (3.1, 10.2)

^a^Includes public non-for profit sector (N = 93)

Availability of malaria blood testing was 94.7% among public health facilities and 68.4% among CHWs. Malaria blood testing stocking rates were largely attributed to the availability of RDT.

The readiness of public-sector outlets for malaria case management, defined as stocking both quality-assured ACT and having malaria blood testing, was 89.0% among public health facilities and 49.7% among CHWs.

### Private sector availability

Among all screened private sector outlets, availability of anti-malarials was as follows: 85.8%, private for-profit facilities; 94.6%, pharmacies; 27.5%, general retailers; and 67.7%, itinerant drug vendors (Table 2).

**Table 2:** Availability of anti-malarial and malaria blood testing among the private outlets

	Private for-profit facility	Pharmacy	General retailer	Itinerant drug vendor	Total private sector^a^
	% (95% CI)	% (95% CI)	% (95% CI)	% (95% CI)	% (95% CI)
Among all screened outlets:	**N** **=** **262**	**N** **=** **176**	**N** **=** **5622**	**N** **=** **632**	**N** **=** **6724**
Availability of any anti-malarial	85.8 (77.6, 91.3)	94.6 (76.5, 99.0)	27.5 (21.9, 34.0)	67.7 (38.8, 87.4)	33.3 (30.2, 36.7)
Among anti-malarial stocking outlets, availability of:	**N** **=** **222**	**N** **=** **170**	**N** **=** **1388**	**N** **=** **468**	**N** **=** **2278**
Quality-assured ACT	36.4 (23.0, 52.2)	90.0 (75.0, 96.5)	35.4 (27.9, 43.8)	34.2 (22.3, 48.4)	36.1 (27.7, 45.5)
Quality-assured AL	35.9 (22.7, 51.6)	89.5 (74.9, 96.1)	35.1 (27.5, 43.6)	34.2 (22.3, 48.4)	35.9 (27.5, 45.3)
Quality ACT with the ‘green leaf’ logo	25.0 (15.5, 37.6)	0.1 (0.0, 0.8)	33.2 (25.7, 41.7)	28.9 (17.1, 44.6)	31.3 (23.0, 41.0)
Non quality-assured ACT	19.3 (13.3, 27.2)	100.0 (–)	12.7 (8.4, 18.9)	13.0 (7.5, 21.6)	14.8 (10.6, 20.3)
Sulfadoxine–pyrimethamine	24.6 (16.2, 35.5)	56.6 (39.5, 72.3)	29.4 (19.8, 41.4)	68.1 (42.9, 85.8)	36.4 (22.1, 53.7)
Oral quinine	70.5 (64.0, 76.3)	67.6 (46.7, 83.2)	34.3 (21.1, 50.5)	60.1 (36.1, 80.1)	42.5 (26.1, 60.7)
Chloroquine	11.1	0.0 (–)	71.3 (54.7, 83.6)	38.4 (19.0, 62.4)	59.2 (39.9, 76.1)
Other non-artemisinin	3.4 (1.3, 8.4)	15.6 (8.6, 26.6)	2.1 (1.3, 3.4)	8.6 (5.7, 12.8)	3.6 (2.1, 6.0)
Oral artemisinin monotherapy	0.0 (–)	0.0 (–)	0.0 (–)	0.0 (–)	0.0 (–)
Artesunate injection	1.5 (0.5, 4.3)	23.9 (14.5, 36.8)	0.0 (–)	0.0 (–)	0.5 (0.2, 0.9)
Artemether injection	28.8 (19.9, 39.7)	70.3 (55.5, 81.8)	0.8 (0.3, 2.2)	0.0 (–)	4.1 (2.9, 5.8)
Quinine injection	82.7 (75.7, 88.0)	30.4 (19.0, 44.9)	1.7 (0.8, 3.7)	0.0 (–)	8.7 (6.4, 11.8)
Among outlets stocking anti-malarials today or within the past 3 months, availability of:	**N** **=** **233**	**N** **=** **170**	**N** **=** **1530**	**N** **=** **496**	**N** **=** **2459**
Any diagnostic test	39.2 (30.6, 48.5)	5.0 (1.4, 16.0)	0.1 (0.0, 0.3)	0.0 (–)	3.3 (2.3, 4.7)
Malaria microscopy	17.9 (8.7, 33.2)	0.0 (–)	0.0 (–)	0.0 (–)	1.5 (0.8, 2.8)
RDT	26.1 (16.8, 38.3)	5.0 (1.4, 16.0)	0.1 (0.0, 0.3)	0.0 (–)	2.2 (1.3, 3.8)

^a^Total private sector includes 32 drug stores

Among the outlets stocking at least one anti-malarial in stock, 36.1% had a quality-assured ACT. This was most commonly available among pharmacies (90.0%) compared to private for-profit facilities, general retailers, and itinerant drug vendors (36.4, 35.4 and 34.2%, respectively). 31.3% of ACTs in the private sector were marked with the ‘green leaf’ logo. Adult quality-assured ACT was available in 24.6% of private-sector outlets. The three child formulations were available in less than 15% of the private sector (Additional file 4).

Chloroquine was available in 59.2% of the private sector followed by oral quinine (42.5%) and SP (36.4%), though there were several differences across outlet types. For example, chloroquine was most commonly stocked by general retailers (71.3%) while SP was most commonly available among itinerant drug vendors (68.1%) and oral quinine was available in 70.5% private for-profit facilities.

### Anti-malarial market share

**Fig. 1:**
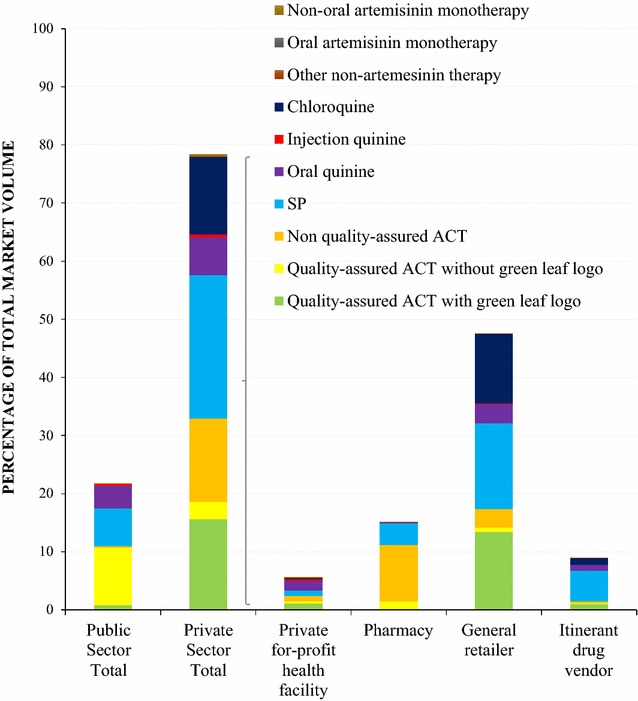
Anti-malarial market share

Figure 1 shows the market share of different categories of anti-malarials sold or distributed in the 7 days prior to the survey. A total of 25,427 anti-malarial AETDs were reportedly distributed in seven days before the survey.

21.5% of the anti-malarial market share was distributed by the public sector, which was comprised mostly of quality-assured ACT without the ‘green leaf’ logo (9.9% of total market share) and of SP (6.5% of the total market).

Almost 80% of the anti-malarials distributed were through the private sector (78.5%). Quality-assured ACT with the ‘green leaf’ logo comprised 15.6% of the total anti-malarial market share, followed by non-quality assured ACT (without the logo), which comprised 14.3%. SP made up the largest market share of non-artemisinin therapies (24.7%), followed by chloroquine (13.3%) and oral quinine (6.5%).

Overall, general retailers dominated the anti-malarial market, accounting for 47.6% of the total market share in Benin, and these providers distributed most of the quality-assured ACT with the ‘green leaf’ logo (13.4% of total market share), SP (14.7%), and chloroquine (12.0%).

### Malaria diagnostic market share

**Fig. 2:**
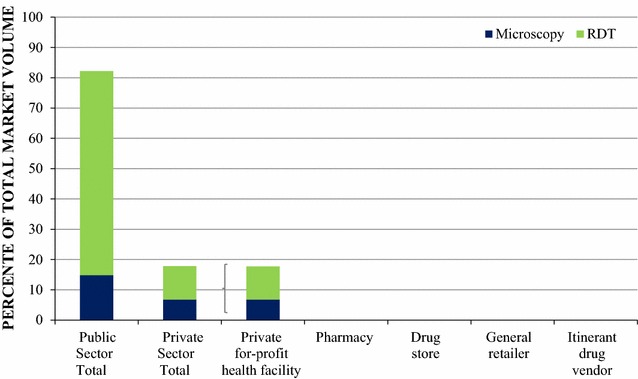
Diagnostic market share

Figure 2 shows the diagnostic market share of different types of malaria tests administered in the seven days prior to the survey. A total of 6712 malaria test units, either microscopy or RDT, were reportedly distributed or used in the seven days prior the outlet survey.

Most of the malaria testing was performed through the public sector, which accounted for 82.2% of the total diagnostic testing market share. Microscopy testing was rare across both the public and the , 14.8 and 6.8% respectively.

Within tprivate sectorhe private sector, malaria blood testing market share was dominated entirely by private for-profit health facilities since none of the other private sector outlets reportedly distributed or sold malaria testing in the seven days before the survey.

### Price

Private sector price of AETD quality-assured ACT ($1.35, inter quartile range [IQR] $1.0, $2.02) was three times more expensive than SP ($0.42, IQR $0.34, $0.51) or chloroquine ($0.41, IQR $0.41–$0.42). The price of AETD quinine was $3.54 (IQR $2.83–$4.25)—2.6 times much more expensive than one quality-assured ACT.

### Provider perceptions of most effective treatment

**Fig. 3:**
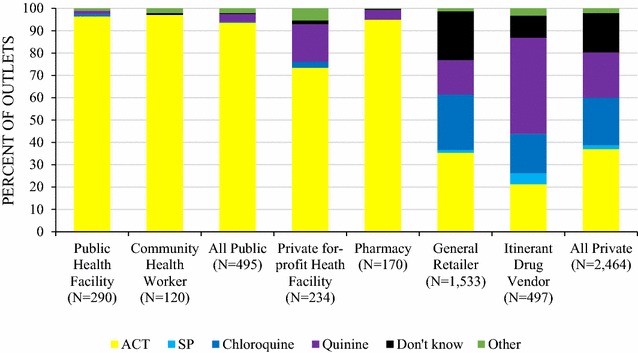
Providers’ perceptions of the most effective treatment for an uncomplicated malaria in a child

**Fig. 4:**
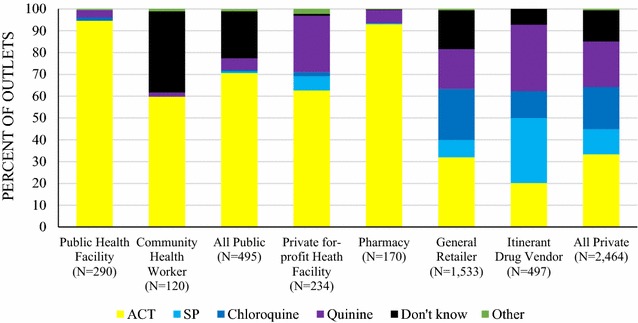
Providers’ perceptions of the most effective treatment for an uncomplicated malaria in an adult

When providers were asked what they perceived to be the most effective anti-malarial for the treatment of uncomplicated malaria in children or adults, results from the public sector illustrate that most providers cited an ACT. Among public health facility providers, 94.6 and 96.4% perceived ACTs was the most effective treatment in adults and in children respectively (Figs. 3, 4). Specific to the question regarding the most effective treatment for adults, 37.2% of CHWs responded that they did not know, while 59.8% perceived ACT as the most effective for an adult and 91.8% of them perceived an ACT as the most effective for children.

In the private sector, most 62.7% of private for-profit and 93% of pharmacy providers cited an ACT as the most effective treatment for adults, and 73.4 and 94.9% respectively cited this as most effective for children. Non-artemisinin therapies, typically chloroquine and quinine, were cited as most effective treatment among general retailers (chloroquine, children: 24.8%; adults: 34.4%; quinine, children: 15.4%; adults: 18.3%) and itinerant drug vendors (chloroquine, children: 17.6%; adults: 29.8%; quinine, children: 43.1%; adults: 30.5%). SP was commonly cited as the most effective treatment for adults by itinerant drug vendors (29.8%).

## Discussion

The 2016 outlet survey provided a complete picture of the malaria testing and treatment landscape across the public and private sectors, providing information on availability, market share, price, and provider perceptions. The findings point to recommendations for improving private-sector malaria case management in Benin.

### Public sector readiness for appropriate malaria case management

Public health facilities showed high readiness for appropriate case management in Benin. There was nearly universal coverage of quality-assured ACT treatment and malaria blood testing in these facilities. These findings reflect national strategies that have been in place since 2011, which stipulate confirmatory testing prior to treatment for all ages and at all levels of care [6]. The current levels of readiness reflect a substantial increase from diagnostic availability measured in 2011, where just over half of the public health facilities had malaria testing available (56.8%) [17], illustrating that national policy has been successful in increasing access to confirmatory testing in this sector.

Three-quarters of public health facilities had SP available for IPTp treatment, reflecting an increase over time, from 17.2% in 2011 and 44.7% in 2014. This suggests substantial progress has been made with regards to the scale-up of SP for IPTp [17, 18]. This is in-line with recent national strategies to increase access to SP, including changes to the dosing regimen, and efforts to provide malaria services free of charge to pregnant women [6]. Availability of oral quinine, recommended for the treatment of uncomplicated malaria in pregnancy during the first trimester, was also high, with over 85% of public health facilities stocking this medicine. These findings illustrate overall readiness among public health facilities to manage malaria in pregnant women.

According to the 2015 national guidelines, injectable quinine followed by oral quinine are still the recommended treatment for severe malaria, which could explain the high levels of quinine availability in public health facilities. However, it is possible that quinine is being used for uncomplicated malaria given it is widely available throughout all types of public health facilities. Quinine should only be administered at hospitals, which would be equipped to manage patients with severe malaria. Furthermore, while a full course of quinine tablets are indicated for treatment of severe malaria, this should only be administered after a primary treatment with injectable quinine. However, market share data illustrate that oral quinine comprises one in every fifth anti-malarial distributed in the public sector, while quinine injection is negligible, suggesting that oral quinine may be routinely administered for uncomplicated malaria. Indeed, a recent household study in southern Benin found quinine was the second most used anti-malarial for self-medication (after ACT) suggesting that efforts are needed to ensure the appropriate administration of this anti-malarial [21]. Despite the updated WHO standards, artesunate availability remains low (5.3%). Efforts are currently underway to identify the barriers to increasing injectable artesunate use for severe malaria treatment in Benin [6].

Since 2014, the reach of the public sector has been extended to the community-level through the training and equipping of CHWs with malaria case management skills and supplies (AL and RDT). Since then, several investments have been made to increase the capacity and coordination of these providers [6]. The results from this survey illustrate how more than half of the CHWs had anti-malarials in stock, namely quality-assured ACT, and almost 70% had RDTs. The availability findings also reflect promising changes from earlier survey rounds where availability of ACT in 2011 was less than 50% and the availability of RDT was negligible (<5%). Furthermore, most CHWs perceived ACT to be the most effective treatment for uncomplicated malaria in adults and children. These findings point to the success of a national level campaign to scale-up, train, and supply CHWs to provide ACT and blood testing services. Key areas to address may be improving CHW awareness of the most effective anti-malarial for adults; given 40% did not know what this was, and to maintain supply of RDTs as a means to increase access to confirmatory testing.

### Role of the private sector in malaria case management

Results from the study confirmed the dominant role of the private sector across Benin, where almost 80% of all anti-malarials passed through this sector, mainly through general retailers—which accounted for almost half of the anti-malarial market share in 2016 (47.6%). Of the 5600 general retail outlets that were screened for anti-malarials, over one in four had anti-malarials in stock, reflecting a three-fold increase from previous surveys [17, 18]. General retailers as a source of anti-malarial treatment have also been documented in other countries, including Madagascar, Myanmar, and Cambodia [22–24], and were also a common source of treatment in Benin as evidenced in a population based survey [25]. The results also point to the importance of itinerant drug vendors, of which over half of those surveyed had anti-malarials available, and comprised around one tenth of the anti-malarial market share. Trend data also illustrate how the combined anti-malarial market share of general retailers and itinerant drug vendors, subsequently referred to as the ‘informal’ private sector, has increased over time from 30.9% in 2011, 40.1% in 2014, to 56.8% in 2016 [17, 18], illustrating the increasing relevancy of these outlets in the delivery of anti-malarial treatment. It is unclear why an increase in the informal market composition has been observed. Given there is little regulation of the private sector in Benin, this growth of the informal sector market composition may reflect a natural evolution of the market to meet consumer demand for anti-malarials, and perhaps these outlets more accessible to patients. In absence of regulation, general retailers and itinerant drug vendors have perhaps responded to consumer demand by stocking anti-malarials in addition to other products.

Given a large portion of the private-sector case management is being channeled through these informal outlets, there may be several opportunities to strengthen the malaria case management services provided by these vendors. There are examples in the literature of innovative strategies that have focused on general retailers and itinerant drug vendors to improve access to quality-assured ACT [24]. There is also a growing body of support for itinerant drug vendors as a means to improve home-based management of malaria [26, 27], and these mobile providers have been cited as a useful means to improve the provision of care for malaria [28]. In Benin, there is also documentation of ‘associations’ of drug vendors, which operate within traditional markets and perform quasi-regulatory functions [11]. The quasi-formal nature of these vendors may make them suitable for accreditation programmes as a means to further regulate, supervise, and engage with the private sector in both ACT and RDT distribution. Such strategies, done in collaboration with the public sector, may help to complement rather than compete with the existing CHW programme. Considering the informal sector in the accreditation process may be an important strategy to accelerate coverage of appropriate case management in Benin.

### Readiness of the private sector in malaria case management

The private sector was generally less well-equipped to test and appropriately treat malaria infections as compared with the public sector. Only one-third of private-sector outlets were stocking quality-assured ACT. Non-artemisinin therapies were more commonly available and distributed. Availability of malaria testing was also negligible and consistent with these findings, most malaria tests were administered by the public sector, which comprised over 80% of the diagnostic market share. Given most private-sector outlets were not stocking malaria tests suggests that presumptive treatment is widespread.

### Availability and market share ACT

While the AMFm or subsequent CPM programme was not implemented in Benin, most of the quality-assured ACT reportedly distributed in the private sector had the AMFm ‘green leaf’ logo. This indicates leakage of anti-malarials’ from other countries and suggests that anti-malarials are being illegally traded into non-subsidized private markets.

The widespread availability and distribution of quality-assured ACT with the logo is perhaps not surprising considering Benin’s supply chain [11]. The domestic anti-malarial market in Benin is relatively small, with few local manufacturers, so the country’s supply relies heavily on imports. Many of the anti-malarial supplies are obtained from more developed pharmaceutical markets in surrounding countries, most notably Nigeria, and imported largely though the informal sector. Thus, it is quite likely that products with the ‘green leaf’ logo—a marker of the subsidized CPM ACT—have leaked into Benin’s private-sector outlets through neighbouring Nigeria. In fact, prior to the AMFm, the importation of medicines illegally from Nigeria was noted as commonplace, with vendors citing ease of accessing cheap suppliers in Lagos as a key reason for the illegal import [11]. The widespread uptake of this illegally imported ACT speaks to the need for a national level programme targeting the private sector with subsidized quality-assured ACT to align the private-sector outlets with national treatment guidelines, as well as strengthen border control and regulation.

Availability and distribution of other non-quality assured ACT was also high, comprising 14.3% of the anti-malarial market and reflecting a slight increase from earlier survey rounds. This is of concern given that non-quality assured ACT medicines have not received pre-qualification, meaning that these medicines have not necessarily been manufactured according to quality standards yielding safe and efficacious medicines. Moreover, non quality-assured ACT have an increased likelihood of being poor quality as evidenced by studies that have tested the pharmacological properties of the medicines [29]. The widespread presence of non-quality assured ACT is of concern given its presence on the market and use poses a threat to appropriate and effective malaria case management.

### Availability of different AL formulations

While the strength of all first-line AL tablets for treatment of uncomplicated malaria is indeed the same, the implementation of the AL policy includes delivery of four different AL pack sizes (6, 12, 18 and 24 tablets) suitable for management of four different weight categories of patients (5–14; 15–24; 25–34 and ≥35 kg). In the private sector, as well as the public sector, availability of the different weight categories was relatively poor. For example, in the private sector, only 11.4% of the private for-profit facilities and 58.6% of pharmacies had AL treatments for children under 5.

Maintaining a consistent supply of age/weight appropriate commodities will be key to ensure that ACT commodities are administered according to the recommended age and weight band of each patient and to prevent medicine packages from being cut or tampered with. This is particularly important given evidence that AL treatment is up to six times more likely to be prescribed if the weight specific pack is in stock [30]. While several strategies are underway to better manage the supply and procurement of malaria commodities to avoid stock-outs, this has not been fully implemented. Temporary options may be to instruct providers to administer AL even if adequate AL pack sizes are not in stock. However, evidence suggests that this practice may compromise high levels of patients’ adherence to AL [31] and incorrect dosing [32, 33]. If adequate availability of first-line ACT treatments cannot be ensured, alternative AL preparations that do not depend on separate packaging, could also be considered [30].

### Availability and use of non-artemisinin therapies

Over a decade after the change in first-line treatment for uncomplicated malaria, non-artemisinin therapies, including SP, oral quinine, and chloroquine, accounted for the majority (57.7%) of the market share in the private sector. SP made up over half of the non-artemisinin therapies reportedly distributed. While most of the SP distribution was through itinerant drug vendors and general retailers, SP was also commonly distributed by pharmacies. The widespread distribution of this medicine implies that it is being used for malaria case management rather than exclusively for IPTp as recommended. Widespread availability and distribution of oral quinine, particularly among general retailers and itinerant drug vendors, also indicates this is being used for the treatment of uncomplicated malaria.

Widespread distribution of non-artemisinin therapies in Benin might be explained by a number of factors. This may in part be attributed to price, given that SP and chloroquine were three times less expensive than quality-assured ACT. Alternatively, access may also be an important factor. Non artemisinin therapies were more widely available than quality-assured ACT—particularly among general retailers where most anti-malarials were distributed. Another reason may be around provider perceptions of the most effective treatment for uncomplicated malaria. In 2016, most of the itinerant drug vendors and general retailers perceived non-artemisinin therapies (SP, chloroquine, or quinine) as the most effective treatment for uncomplicated malaria.

To improve private-sector case management, removal of non-artemisinin therapies from the market is paramount and new strategies are necessary to curtail their consumption and promote the use of quality-assured ACT and RDT in the private sector. Several programmes have been implemented across sub-Saharan Africa to improve private sector readiness for appropriate malaria case management that could be relevant in the Benin context. A similar nation-wide subsidy to that of the AMFm may be an immediate means to overcome ACT access and affordability issues for this treatment, as evidence by the pilot initiative [34, 35]. Once barriers related to access of quality-assured ACT have been addressed, mass-media behaviour change campaigns may be a particularly effective strategy in Benin to increase awareness of the first-line treatment and to promote demand for the quality ACT product. Several studies have demonstrated how consumer demand is associated with treatment and how patient preferences influence provider dispensing behaviour [36–39]. Specifically in Benin, qualitative research found that provider stocking decisions were overwhelmingly driven by patient demand, which led some outlets not to stock ACT [11]. Furthermore, provider training and supervision may also be merited to improve the quality of case management practices, including accreditation of outlets as previously discussed. Such multi-pronged strategies are likely to improve malaria case management and can improve private sector readiness and performance, as has been demonstrated in other contexts [12].

### Availability of oral artemisinin monotherapy

Oral artemisinin monotherapy poses a serious threat to the continued efficacy of artemisinins, and as such this anti-malarial was banned in Benin in 2008. In 2016, no oral artemisinin monotherapy was detected in the market. This is of promise given ACTwatch outlet survey findings from neighboring Nigeria which show that availability of oral artemisinin monotherapy in the private sector has increased from 24.6% in 2013 to 37.3% in 2015 [40]. Furthermore, in the north-west of the country—in areas close to the border of Benin—availability of oral artemisinin monotherapy was even higher at 43.3%. Given that Nigeria appears to be a source of supply of anti-malarials to Benin’s private sector market, it is important that availability of oral artemisinin monotherapy in the market is routinely monitored. Mystery clients to detect unwanted or banned medicines may be a useful method to do this [41].

## Limitations

The ACTwatch outlet survey design has limitations that have been documented and reported [9, 15, 19]. One point to mention is that while anti-malarial audits were carried out by researchers, sales volumes were reported by the provider and these responses were open to positive response bias. The pros and cons of using self-reported sales volumes, versus other methods to capture market share such as sale inventory audits or exit interviewers, suggests that there are advantages and disadvantages of different methods but no method is gold standard and each has its own limitations [42].

Other specific limitations to Benin’s outlet survey include the use of two different forms of data collection (electronic and paper questionnaires). While electronic data collection has the advantage of recording the data instantly with all the relevant checks and skip patterns built into the programme, it may have had an impact on respondents’ fear that they were being recorded or investigated. In addition, some itinerant vendors could have been missed during the survey given these vendors may work late at night and, for security reasons, interviewers only worked during the day and early evening.

## Conclusions

The public sector in Benin is typically well equipped to test and appropriately treat malaria according to national treatment guidelines. However, the private sector is responsible for most of the anti-malarial distribution, typically through general retailers, and this channel most commonly distributes non-artemisinin therapies. There is also evidence of leakage of subsidized ACT from neighbouring countries. A national strategy to scale up access to first-line, quality-assured, subsidized treatment as a means to improve coverage and quality of malaria case management services is needed. Strategies to increase coverage of malaria commodities should be supported by interventions to address provider perceptions, as well as consumer behaviours, and innovative approaches to either engage or regulate Benin’s informal private sector are needed.

## Additional files

**Additional file 1.** Sampling weights.

**Additional file 2.** Detailed sample description.

**Additional file 3.** Availability of quality-assured AL among all screened public sector outlets.

**Additional file 4.** Availability of quality-assured AL among anti-malarial stocking private outlets.

## Abbreviations

ABSM: Association Beninoise pour le Marketing Social
ACT: artemisinin-based combination therapy
AL: artemether–lumefantrine
AETD: adult equivalent treatment dose
AMFm: affordable medicines facility for malaria
CHW: community health worker
CFA: Communauté Financière Africaine
CPM: co-payment mechanism
IPTp: intermittent treatment as prevention during pregnancy
IQR: inter quartile range
NMCP: National Malaria Control Programme
RDT: rapid diagnostic test
WHO: World Health Organization
SP: sulfadoxine–pyrimethamine
USA: United States of America
