# Supplementary material for: The malaria testing and treatment landscape in Benin
Source: Malar J. 2017 Apr 26;16:174. doi: 10.1186/s12936-017-1808-x (PMC5405537; doi:10.1186/s12936-017-1808-x)
Supplement: Supplementary file 1 — Additional file 1. Sampling weights. [file 12936_2017_1808_MOESM1_ESM.docx]

Sampling weights were applied for analysis of the Benin 2014 outlet survey data to account for variations in probability of selection as a result of the sampling design:

1) **Stratification**: Disproportionate allocation stratification was used to ensure adequate sample size within the urban and rural domains to allow for domain-specific estimates. The research domains were based on national designation of urban and rural wards. A representative sample was selected within each domain.

2) **One-stage cluster sampling**: Arrondissements were selected from sampling frames within each domain with probability proportional to size. Within each arrondissement, a census of all outlets with the potential to sell or distribute antimalarials and/or provide malaria blood testing was conducted.

3) **Booster sample**: The geographic area for the outlet census was extended to the commune level for public health facilities, pharmacies and drug stores. All public health facilities, pharmacies and drug stores within communes in which the selected arrondissements were located were included in the study.

The sampling weights applied during analysis are the inverse of the probability of selection:

𝑊𝑖=1a × MαΣMα

Where:

Mα = estimated cluster (population size)

ΣMα = sum of estimated cluster sizes (population size) in the entire stratum

a = number of clusters selected within the stratum

Sampling weights are calculated at the cluster level and are applied to all outlets within a given cluster, irrespective of outlet type.

Market share was calculated using the full census data at the arrondissement level only (i.e. the booster sample was not included in market share calculations). Arrondissement sampling weights were created using the sampling weight formula (*Wi*), where:

Mα = estimated arrondissement population size

ΣMα = sum of estimated arrondissement population size in the entire stratum

a = number of arrondissements selected within the stratum

The arrondissement sampling weights were applied to all other indicators in the report for all outlet types with the exception of:

1. Public health facilities, pharmacies and drug stores: Given that these outlet types were included in the sample through a commune-wide census, the weights applied to these outlet types for all indicators other than market share were calculated using the sampling weight formula (*Wi*), where:

Mα = estimated commune population size

ΣMα = sum of estimated commune population size in the entire stratum

a = number of communes selected within the stratum
