# Supplementary material for: The malaria testing and treatment landscape in Benin
Source: Malar J. 2017 Apr 26;16:174. doi: 10.1186/s12936-017-1808-x (PMC5405537; doi:10.1186/s12936-017-1808-x)
Supplement: Supplementary file 2 — Additional file 2. Detailed sample description. [file 12936_2017_1808_MOESM2_ESM.docx]

**Additional File 2: Detailed sample description.**

|  | Public  Health  Facility | CHW | Private not for-profit  facility | Private  for-profit  facility | Pharmacy | Drug store | General retailer | Itinerant drug vendor | **Total** |
| --- | --- | --- | --- | --- | --- | --- | --- | --- | --- |
| **Number of outlets screened** | 298 | 145 | 93 | 262 | 176 | 32 | 5622 | 632 | 7260 |
| Census | 72 | 145 | 93 | 262 | 60 | 9 | 5622 | 632 | 6895 |
| Booster | 226 | 0 | 0 | 0 | 116 | 23 | 0 | 0 | 365 |
| **Number of outlets eligible and interviewed** | 290 | 121 | 87 | 238 | 170 | 30 | 1533 | 497 | 2966 |
| Census | 68 | 121 | 87 | 238 | 58 | 8 | 1533 | 497 | 2610 |
| Booster | 222 | 0 | 0 | 0 | 112 | 22 | 0 | 0 | 356 |
| **Number of outlets eligible but not interviewed** | 1 | 1 | 0 | 10 | 6 | 1 | 15 | 1 | 35 |
| Census | 0 | 1 | 0 | 10 | 2 | 0 | 15 | 1 | 29 |
| Booster | 1 | 0 | 0 | 0 | 4 | 1 | 0 | 0 | 6 |
| **Number of interviewed outlets with at least one anti-malarial in stock on the day of the survey** | 286 | 91 | 83 | 222 | 170 | 30 | 1388 | 468 | 2738 |
| Census | 65 | 91 | 83 | 222 | 58 | 8 | 1388 | 468 | 2383 |
| Booster | 221 | 0 | 0 | 0 | 112 | 22 | 0 | 0 | 355 |
| **Number of interviewed outlets with at least one anti-malarial in stock on the day of the survey or at least one anti-malarial reportedly in stock in the previous 3 months** | 290 | 120 | 85 | 234 | 170 | 30 | 1533 | 497 | 2959 |
| Census | 68 | 120 | 85 | 234 | 58 | 8 | 1533 | 497 | 2603 |
| Booster | 222 | 0 | 0 | 0 | 112 | 22 | 0 | 0 | 356 |
| **Number of interviewed outlets that provide malaria blood testing, but do not stock anti-malarial medicines** | 0 | 1 | 2 | 4 | 0 | 0 | 0 | 0 | 7 |
| Census | 0 | 1 | 2 | 4 | 0 | 0 | 0 | 0 | 7 |
| Booster | 0 | 0 | 0 | 0 | 0 | 0 | 0 | 0 | 0 |
| **Number of interviewed outlets that reported distributing anti-malarials in the week prior to the survey** | 269 | 69 | 68 | 200 | 131 | 29 | 1217 | 455 | 2438 |
| Census | 60 | 69 | 68 | 200 | 44 | 8 | 1217 | 455 | 2121 |
| Booster | 209 | 0 | 0 | 0 | 87 | 21 | 0 | 0 | 317 |
| **Number of interviewed outlets that reported providing/distributing a malaria diagnostic test in the week prior to the survey** | 238 | 75 | 33 | 89 | 3 | 0 | 1 | 0 | 439 |
| Census | 55 | 75 | 33 | 89 | 0 | 0 | 1 | 0 | 253 |
| Booster | 183 | 0 | 0 | 0 | 3 | 0 | 0 | 0 | 186 |
