# Supplementary material for: The malaria testing and treatment landscape in Benin
Source: Malar J. 2017 Apr 26;16:174. doi: 10.1186/s12936-017-1808-x (PMC5405537; doi:10.1186/s12936-017-1808-x)
Supplement: Supplementary file 3 — Additional file 3. Availability of quality-assured AL among all screened public sector outlets. [file 12936_2017_1808_MOESM3_ESM.docx]

**Additional File 3: Availability of quality-assured AL among all screened public sector outlets**

|  | **Public**  **Health Facility** | **CHW** | **Total**  **Public Sector** |
| --- | --- | --- | --- |
|  | %  (95% CI) | %  (95% CI) | %  (95% CI) |
| **Quality-assured AL**  **for children:** | N=298 | N=145 | N=536 |
| 20/120 pack 6 | 65.9 | 50.4 | 48.2 |
|  | (56.3, 74.3) | (29.1, 71.5) | (34.5, 62.3) |
| 20/120 pack 12 | 58.1 | 4.7 | 18.7 |
|  | (47.5, 68.0) | (1.4, 14.4) | (12.5, 26.9) |
| 20/120 pack 18 | 48.8 | 0.6 | 13.3 |
|  | (39.3, 58.4) | (0.1, 4.1) | (9.1, 19.0) |
| **Quality-assured AL**  **for an adult:** | N=298 | N=145 | N=536 |
| 20/120 pack 24 | 57.2 | 1.7 | 17.8 |
|  | (46.0, 67.6) | (0.5, 6.1) | (12.1, 25.4) |
| 80/480 pack 6 | 0.0 | 0.0 | 0.0 |
