# Supplementary material for: The malaria testing and treatment landscape in Benin
Source: Malar J. 2017 Apr 26;16:174. doi: 10.1186/s12936-017-1808-x (PMC5405537; doi:10.1186/s12936-017-1808-x)
Supplement: Supplementary file 4 — Additional file 4. Availability of quality-assured AL among anti-malarial stocking private outlets. [file 12936_2017_1808_MOESM4_ESM.docx]

**Additional File 4: Availability of quality-assured AL among anti-malarial stocking private outlets**

|  | **Private**  **for-profit facility** | **Pharmacy** | **Drug**  **store** | **General** retailer | **Itinerant drug**  **vendor** | **Total Private Sector **** |
| --- | --- | --- | --- | --- | --- | --- |
|  | %  (95% CI) | %  (95% CI) | %  (95% CI) | %  (95% CI) | %  (95% CI) | %  (95% CI) |
| **Quality-assured AL**  **for children:** | **N=222** | **N=170** | **N=30** | **N=1,388** | **N=468** | **N=2,278** |
| 20/120 pack 6 | 11.4 | 58.6 | 6.1 | 12.3 | 6.5 | 11.8 |
|  | (5.4, 22.4) | (43.1, 72.5) | (1.4, 22.3) | (8.6, 17.3) | (2.5, 15.8) | (7.8, 17.4) |
| 20/120 pack 12 | 10.7 | 40.8 | 17.5 | 14.9 | 8.0 | 13.7 |
|  | (6.6, 16.7) | (25.3, 58.4) | (4.5, 48.7) | (10.7, 20.3) | (3.6, 17.0) | (9.2, 20.0) |
| 20/120 pack 18 | 5.7 | 0.1 | 16.2 | 4.6 | 2.8 | 4.4 |
|  | (2.4, 12.8) | (0.0, 0.8) | (2.5, 59.0) | (2.9, 7.2) | (0.8, 9.1) | (2.7, 7.1) |
| **Quality-assured AL**  **for an adult:** |  |  |  |  |  |  |
| 20/120 pack 24 | 21.1 | 85.6 | 33.8 | 23.8 | 24.3 | 24.6 |
|  | (12.8, 32.6) | (72.0, 93.3) | (14.5, 60.7) | (18.0, 30.7) | (17.0, 33.6) | (18.8, 31.4) |
| 80/480 pack 6 | 0.0 | 37.9 | 0.0 | 0.0 | 0.0 | 0.5 |
|  | - | (25.0, 52.9) | - | - | - | (0.3, 0.9) |
